# The Work of the Imperial Cancer Research Fund.—Part I

**Published:** 1905-09-02

**Authors:** 


### Hospital Clinics.

### THE WORK OF THE IMPERIAL CANCER RESEARCH FUND.—Part I.

Two further reports upon the work done by the officers of the above fund have recently been published. They take the shape of two blue-books, Part I. dealing with the statistical investigation of cancer,* Part II. with the growth of cancer under natural and experimental conditions. Both volumes contain matter of the highest importance, a brief summary of which will be provided in these columns.

The volume dealing with the statistical investigation of cancer [1] enters a warning as to the natural limitations of this proceeding, limitations prescribed by the difficulty of obtaining accurate information. No matter how careful the preparation of statistics from an actuarial point of view, if the reports upon which they are based are inaccurate, all deduction will be equally so. For instance, cancer-censuses have been carried out in Germany and elsewhere. Such a census implies the enumeration on a given day of all cases of malignant new-growth under the observation of medical practitioners throughout the country. But such a census does not supply the facts with anything approaching accuracy. It supplies an imperfect count of cases diagnosed as cancer, the fallacies of which proceeding, as checked by more complete hospital returns, will be alluded to at a later period. In view of these fallacies and of the failure of cancer-censuses in other countries to advance knowledge of the disease, Dr. Bashford and his colleagues decided not to advise a similar census in this country, but to accumulate statistics from sources of narrower limitation but of infinitely greater accuracy. These sources exist in a number of the larger metropolitan hospitals, and a card-index system has been instituted which provides all the necessary information upon uniform lines.

The report insists upon the necessity for a microscopic corroboration of the diagnosis of cancer. Inquiries into the relation between the results of clinical and gross post-mortem examination on the one hand, and microscopical examination on the other proved that the former two, considered alone, were a fruitful source of fallacy. For the purposes of the Fund-statistics therefore, separate groups have been made to embrace cases in which a microscopic examination was made and those in which it was omitted. Further, a preliminary examination of hospital records showed that the frequency of microscopical investigation varied with the site of the tumour, those tumours which are most accessible to physical examination being most frequently so investigated. In all situations the histological examination revealed important errors in diagnosis, but the error was greatest when the tumours were most inaccessible. Three anatomical groups have therefore been instituted for purposes of statistical convenience. A, accessible; B, inaccessible to careful physical examination; and C, an intermediate group.

* *Note.*—The word "cancer" is used in the report as a synonym for malignant new-growth generally.

In order to determine what may be called the "normal" error in diagnosis a search was made in hospital records during 1903. A total of 866 new growths, of which microscopical records existed, showed that in 683 cases a correct diagnosis of malignancy was reached, in 152 cases the growth was malignant but undiagnosed as such, while in 31 a diagnosis of malignancy was discredited by the microscope. These figures sufficiently emphasise the fallacies vitiating any but the most careful statistics. They are borne out moreover by subsequent tables based upon the card-index reports adopted by the fund; these latter tables show that of the accessible cases some 8 per cent. go undiagnosed, the percentage rising to 38 in the case of inaccessible tumours. The error in diagnosis is higher among children than among adult patients.

It has long been held that sarcoma was a malignant process peculiarly affecting the young, but the investigations of the fund prove that this is not so. It has been found from an analysis of the hospital cases that malignant tumours in certain selected sites are invariably sarcomatous. These sites are the globe of the eye, the orbit, the arm and leg, the pelvic bones and the buttock. On the assumption that malignant growths in these situations are sarcomatous, the returns of the Registrar-General show that the frequency of sarcoma increases with age in the manner long known to be characteristic of carcinoma. This result indicates the important conclusion that sarcomata and carcinomata are manifestations in different tissues of a process which is essentially the same. If this be so it is clear that their points of similarity, rather than their points of difference, are likely to throw light upon the fundamental phenomena of either.

Carcinoma and sarcoma, therefore, both occur with increasing frequency as age advances. The national figures show that the incidence of malignant new growths among the same number of persons is ten times as great at the age of seventy as it is at the age of thirty. The neglect of this fact has vitiated many statistical investigations designed to prove the endemic character of the disease in certain geographical districts. This added frequency suggests a correlation between cancer and the periods of decline in reproductive activity. But the occurrence of cancer in castrated and spayed animals at the same ages as in entire animals shows that neither an abnormal internal secretion in the reproductive organs, nor the cessation of a normal one, is the cause of cancer.

The same rule of age-incidence which is observed in cancers of men is found to hold in the case of animals. Whenever aged cows are slaughtered in large numbers cancer is not infrequently discovered during inspection of the carcases. Among 62,935 home cattle of all ages slaughtered at Glasgow malignant new-growth with extensive dissemination was discovered in 27, all the latter being aged cows imported from Ireland. In the following year 131 malignant new-growths were found among 43,362 carcases, 116 of the former again occurring in aged cows from Ireland. The question of age, therefore, acquires an overwhelming importance in any inquiry into the local frequency of cancer. In all domesticated animals the age at which malignant new-growths make their appearance stands in a direct relation to the normal duration of life in each species, so that the ratio of the periods of relative freedom from cancer to the normal duration of life remains the same for all the observed varieties of animal. But not only has each individual the normal age limit of his species. The cellular elements of different organs have their own mean duration of activity, and cancer affects these organs in their respective senility. Thus the chorion has a short life, and chorion-epithelioma appears at an interval after fertilisation which corresponds to the declining age of the chorion. The mamma and the uterus live longer, but the maximum incidence of cancer upon them is at the era of their physiological decline. The skin remains functional long after middle life, and the greatest frequency of squamous-celled carcinoma is correspondingly deferred.

Inquiry has been made as to the alleged discrepancy between the frequency of cancer in England and Ireland respectively. Returns appear to show that while of the admissions to metropolitan hospitals the proportion of cancer is as 1 to 20, in Irish hospitals it is as 1 to 36. The conclusion arrived at by the Fund investigators is that the discrepancy is artificial and depends upon the infrequency with which post-mortem examinations and microscopical examinations are conducted in Ireland as compared with London.

As regards the ethnological distribution of cancer the available data are not yet sufficient for detailed deduction. None the less, they show that cancer occurs among all the races and under all the climates of the empire. Nor does diet exercise any marked influence upon the occurrence of the disease. Both in the case of animals and man, the carnivorous and vegetarian alike supply victims for the malady.

The general conclusion drawn from the records so far supplied to the staff of the Fund is that, if England be taken as a standard, the precision with which cancer is identified progressively diminishes throughout the empire, and thus the extent to which it is recorded is inversely proportional to the difficulties to be overcome in obtaining information. This apparent platitude cannot be neglected, for it supplies what is probably the correct interpretation of the alleged increase in the occurrence of cancer. The data tend to show that this increase is not real, for they show that malignant new-growths undoubtedly attack fishes, that they attack the lower mammals with a frequency far greater than was previously believed to be the case, in fact, that the reputed increase depends upon an enhanced precision in the matter of diagnosis.

It has been said that the death-rate from cancer progressively diminishes as younger age-periods come into review. It is important to note that this diminution does not extend backwards uniformly to birth. The minimum is reached between the fifth and the fifteenth years, the curve rising again as the date of birth is more nearly approached. There is hitherto no explanation of this tendency.

Part I. concludes with a series of useful tables of accurate statistics from various sources throughout the empire. The whole volume gives evidence of accurate and laborious work, but it is regrettable that greater lucidity and coherence has not been attained in the arrangement of the varying sections of which it is made up.

[1] Scientific reports of the Imperial Cancer Research Fund. Part I. London. 1905. Price 2s. 6d.

